# Active Modulating the Intensity of Bifocal Metalens with Electrically Tunable Barium Titanate (BTO) Nanofins

**DOI:** 10.3390/nano11082023

**Published:** 2021-08-08

**Authors:** Shuai Qin, Hui Huang, Kaiqian Jie, Sirui Zeng, Li Chen, Hongzhan Liu, Jianping Guo, Hongyun Meng, Faqiang Wang, Xiangbo Yang, Zhongchao Wei

**Affiliations:** 1 Guangdong Provincial Key Laboratory of Nanophotonic Functional Materials and Devices, School of Information and Optoelectronic Science and Engineering, South China Normal University, Guangzhou 510006, China; shuaiqin@m.scnu.edu.cn (S.Q.); huihuang@m.scnu.edu.cn (H.H.); kqjie@m.scnu.edu.cn (K.J.); lichen@m.scnu.edu.cn (L.C.); lhzscnu@163.com (H.L.); guojpgz@163.com (J.G.); hymeng@scnu.edu.cn (H.M.); fqwang@scnu.edu.cn (F.W.); xbyang@scnu.edu.cn (X.Y.); 2 School of Civil Engineering, Zhengzhou University, Zhengzhou 450001, China; brightgalahad@gmail.com

**Keywords:** bifocal metalens, barium titanate, electro-optic

## Abstract

The multifocal metalens with an adjustable intensity has great potential in many applications such as the multi-imaging system, but it is less studied. In this paper, by combining the electro-optic material barium titanate (BTO) with the Pancharatnam-Berry phase, an electrically modulated bifocal metalens in a visible light band is innovatively proposed. Due to the electro-optic effect, we can control the refractive index of the BTO nanofins to vary between 2.4 and 3.07 by applying different voltages (0–60 V). Thus, the method of modulating the intensity ratio of the two focal points is applying an electric field. It is different from using phase change materials or changing the ellipticity of incident light, the strategies proposed in previous studies. Moreover, when the applied voltage is 0 V or 60 V, the bifocal metalens becomes a single focal metalens with different focal lengths, and the full width at half maximum of each focal point is close to the diffraction limit. It has great potential in applications of optical storage, communication and imaging systems.

## 1. Introduction

In the past decades, metalens has drawn intensive attention due to the ability to control the wavefront to focus with a small footprint based on methods of realizing the discontinue phase [1,2,3,4,5,6,7,8,9,10]. However, tremendous researches mainly concentrated on focusing with one focal point; the bifocal metalens or multifocal metalens with great potential in multi-imaging and micro-manipulating optics is less studied. With arrays of inhomogeneous optical scatters placed on one or more thin surfaces, the bifocal metalens allows one beam of incident light to focus at two different focal points [11,12,13,14,15]. As a member of metalens, the bifocal metalens also exists with the problem of being static in nature. Therefore, the modulation of bifocal metalens is also a significant topic, especially the tunable intensity in focal points.

Up to now, methods to realize the adjustable intensity of the bifocal metalens have been limited. One important method is combining thermally controlled phase change materials with the bifocal metalens [16,17,18]. Among them, Ge_2_Sb_2_Te_5_ (GST) is the most representative material for the application of the adjustable bifocal metalens. GST is a kind of phase change material that has a significant refractive index shift and absorption difference in different states [19,20,21]. Most of the methods based on GST rely on dividing the metalens into different areas. The design will lead to a low signal-to-noise ratio [17,22]. Moreover, the fractional crystallization of GST is difficult to control [23] which impedes the development of a continuous precise regulation. Additionally, GST is not applicable to a metalens in the visible band with a large absorption in both states. Another phase change material used in the bifocal metalens is VO_2_ which works in the infrared band [24] and terahertz [18]. Similar to GST, it is also not suitable for the realization of the metalens in the visible band.

Except for phase change materials, a major method to modulate the bifocal metalens is altering the polarization of the incident beam [25,26,27,28]. For instance, Shengnan Tian et al., proposed a dielectric longitudinal bifocal metalens with an adjustable intensity [26]. By entering different elliptically polarized light, the method can realize adjusting the relative intensity of the two focal points flexibly. However, the change of incident light may bring inconvenience in some conditions and the response time is not fast enough. An adjustable intensity, high-speed modulation and constant light source are the ideal scenarios for the bifocal metalens in practical application.

**Figure 1:**
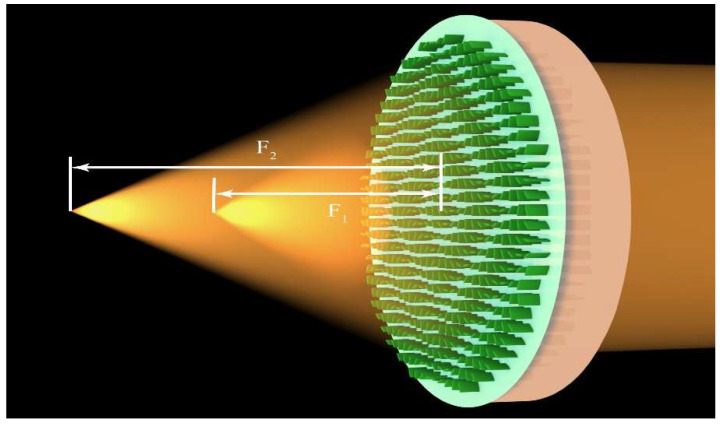
Schematic of the electrically modulated bifocal metalens. The metalens can focus at two different focal points at the same time. By applying different voltages, the intensity ratio of the two focal points changes.

In this paper, we propose an electrically modulated bifocal metalens whose intensity can be adjusted based on the electro-optical material barium titanate (BTO) in the visible region with high-speed modulation. Due to the Pockels effect, the refractive index of BTO can be modulated by varying the applied voltage [29]. Moreover, BTO fits the visible region that can make up for the problem of the working region of phase change materials. As shown in Figure 1, by designing a metalens doublet to combine the multilayer Pancharatnam-Berry phase with BTO, the relative intensity ratio of the two foci can be adjusted. The double-layer structure can realize the unified control of the metalens. When adjusting the intensity, the metalens just needs one applied voltage. It effectively avoids the complexity of applying different controls simultaneously on a micro–nano structure. The upper layer of the metalens is composed of BTO nanofins. The bottom layer is composed of Si nanofins. Additionally, because of the thin thickness between the two layers, to simplify the production in reality, the Si nanofins layer is filled with SiO_2_. In order to realize the arbitrary intensity ratio, the intensity of each focus should be able to change between 0 and the maximum value. The numerical simulations are completed based on the commercial software finite-difference time-domain (FDTD) Solutions 2018a (Lumerical Inc., Vancouver, BC, Canada). The results show that the metalens can focus on a single focal point when *n* = 2.4 (F_1_ = 9 μm) and *n* = 3.07 (F_2_ = 15 μm) to realize the varying of the focal length. The simulated focal lengths correspond to the theoretical values well. Additionally, the full width at half maximums (FWHMs) are close to the diffraction limited values. Then, we gradually increase the refractive index of BTO from 2.4 to 3.07. Values of the intensity ratio between the two foci show that the design has the ability to modulate the metalens flexibly. Additionally, simulated focal lengths and FWHMs show that the image quality under the bifocal condition is still high. We believe the proposed method will provide a potential platform for multi-imaging systems, optical free-space communication and optical data storage.

## 2. Materials and Methods

The metalens is composed of BTO nanofins layer, Si nanofins layer, indium tin oxide (ITO) layer and SiO_2_ substrate. As a kind of electro-optic crystal, BTO has an excellent ability to alter the refractive index. The shift of the refractive index is proportional to applied electric field because of the electro-optic effect named Pockels effect. Meanwhile, BTO is chemically and thermally stable and has ultrafast modulation speed (sub-ps) [29,30]. Additionally, compared with other electric-optical materials such as LiNbO_3_ [31], the Pockels coefficient of BTO is much larger. Therefore, for the same refractive index shift, the applied electric field on BTO is lower. Moreover, its working bandwidth can cover visible and near-infrared region that solves the absorption problem of phase change materials in visible region. The ordinary refractive index of BTO will shift with the application of the voltage as follows [32,33]:

(1) $$n=n_{0}+\frac{1}{2}n_{0}^{3}r_{51}V/t_{D}$$

where n_0_ is the real part of the refractive index of BTO with no application of electric field, the electro-optic (EO) coefficient r_51_ is 1300 pm/V according to the past research. V is the applied voltage to produce refractive index shift and t_D_ is thickness of the BTO layer which means the height of BTO nanofins. In the proposed design, the height of BTO nanofins was set as 800 nm. Therefore, by applying a voltage that was lower than 60 V, the refractive index can shift from 2.4 to 3.07. The function of the ITO layer was to apply voltage from the bottom of BTO layer which makes the metalens easy to be compacted. Moreover, as shown in Figure 1, if the thickness between the Si layer and BTO layer has a relatively large value, it will influence the phase distribution. Therefore, the thickness was set as 0.4 μm, a little larger than half of the wavelength to obtain a better result. In order to avoid the manufacturing problem due to the thickness being so thin, SiO_2_ filled in the Si layer as filling material [34].

**Figure 2:**
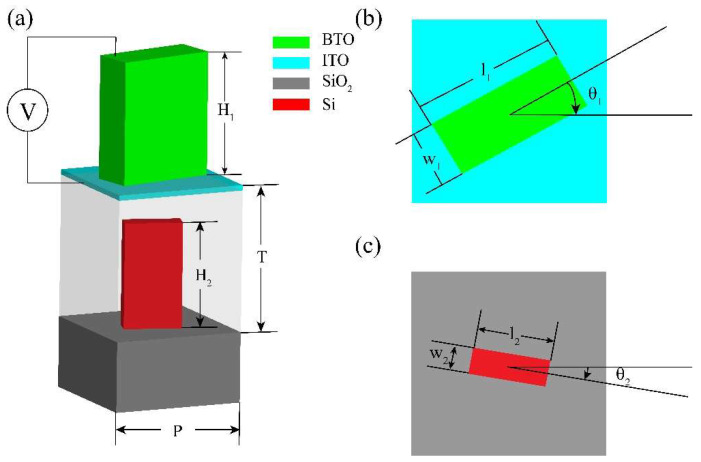
(**a**) Diagrams of the unit cell, the upper layer is BTO nanofin and the bottom layer is Si nanofin. (**b**,**c**) represent the schematic diagrams of the upper layer and lower layer rotation angles. The parameters of BTO nanofin and Si nanofin are l_1_, w_1_, H_1_ and l_2_, w_2_, H_2_, respectively. θ_1_ and θ_2_ are rotation angles of BTO nanofin and Si nanofin. P is the period of the unit cell. T is the distance between the two layers.

As shown in Figure 2, the unit cell is a double-layer structure. According to multilayer Pancharatnam-Berry (PB) phase method, when a circularly polarized beam incidents to the cell, the Jones matrix can be expressed as [34]:

(2) $$J(\theta_{1},\theta_{2})=\left[\begin{array}{cc} \frac{1}{4}T_{1}T_{1}^{\prime }+\frac{1}{4}T_{2}T_{2}^{\prime }e^{j2(\theta_{1}-\theta_{2})} & \frac{1}{4}T_{2}T_{1}^{\prime }e^{j2\theta_{1}}+\frac{1}{4}T_{1}T_{2}^{\prime }e^{j2\theta_{2}}\\ \frac{1}{4}T_{2}T_{1}^{\prime }e^{-j2\theta_{1}}+\frac{1}{4}T_{1}T_{2}^{\prime }e^{-j2\theta_{2}} & \frac{1}{4}T_{1}T_{1}^{\prime }+\frac{1}{4}T_{2}T_{2}^{\prime }e^{j2(\theta_{2}-\theta_{1})} \end{array}\right]$$

(3) $$\{\begin{array}{cc} t_{o}+t_{e}=T_{1} & t_{o}-t_{e}=T_{2}\\ t_{o}{}^{\prime }+t_{e}{}^{\prime }=T_{1}{}^{\prime } & t_{o}{}^{\prime }-t_{e}{}^{\prime }=T_{2}{}^{\prime } \end{array}$$

in each unit cell doublet, θ_1_ and θ_2_ are rotation angles of BTO nanofin and Si nanofin. t_o_, t_e_ (BTO nanofin) and t_o_′, t_e_′ (Si nanofin) represent the complex transmission coefficients along ordinary (l_1_, l_2_) and extraordinary (w_1_, w_2_) axes. For the proposed metalens, the incident beam was right-handed circularly polarized. Therefore, the output beam follows the result:

(4) $$E_{\mathrm{out}}=\frac{1}{4}T_{1}T_{1}^{\prime }\left(\begin{array}{c} 1\\ i \end{array}\right)+\frac{1}{4}T_{2}T_{2}^{\prime }e^{j2(\theta_{1}-\theta_{2})}\left(\begin{array}{c} 1\\ i \end{array}\right)+\frac{1}{4}T_{2}T_{1}^{\prime }e^{-j2\theta_{1}}\left(\begin{array}{c} 1\\ -i \end{array}\right)+\frac{1}{4}T_{1}T_{2}^{\prime }e^{-j2\theta_{2}}\left(\begin{array}{c} 1\\ -i \end{array}\right)$$

In order to realize the condition that the relative intensity ratio between the two foci can cover all values, the metalens should have the ability to focus on one point for extreme condition. It means the ratio can be close to 1/0 or 0/1. First, the Si nanofin works as a half-wave plate which means $T_{1}^{\prime }$ = 0 to decrease the resulting phase distributions:

(5) $$E_{\mathrm{out}}=\frac{1}{4}T_{2}T_{2}^{\prime }e^{j2(\theta_{1}-\theta_{2})}\left(\begin{array}{c} 1\\ i \end{array}\right)+\frac{1}{4}T_{1}T_{2}^{\prime }e^{-j2\theta_{2}}\left(\begin{array}{c} 1\\ -i \end{array}\right)$$

Based on the simplified equation, the function of the BTO nanofin has to be able to switch between a half-wave plate ($T_{1}^{}$ = 0) and a full-wave plate ($T_{2}^{}$ = 0) when the refractive index switches between 2.4 and 3. Therefore, when *n* = 2.4 and 3.07, the output light follows the equation:

(6) $$\{\begin{array}{c} E_{n=2.4}=\frac{1}{4}T_{2}T_{2}^{\prime }e^{j2(\theta_{1}-\theta_{2})}\left(\begin{array}{c} 1\\ i \end{array}\right)\\ E_{n=3.07}=\frac{1}{4}T_{1}T_{2}^{\prime }e^{-j2\theta_{2}}\left(\begin{array}{c} 1\\ -i \end{array}\right) \end{array}$$

Above all, in this paper, when there was no application of voltage, the BTO nanofin worked as a half-wave plate with the imparted phase distribution of the output light equaling 2(θ_1_ − θ_2_). After applying voltage until the refractive index reached 3.07, the inserted phase distribution became −2θ_2_. When the refractive index was between 2.4 and 3.07, the phase distribution of the outgoing light was divided into two parts. One part was E_n_ = 2.4, the other part was E_n_ = 3.07. Additionally, as the refractive index changes, the ratio of the two inserted phase distributions will also change.

## 3. Optimization of Nanofins

**Figure 3:**
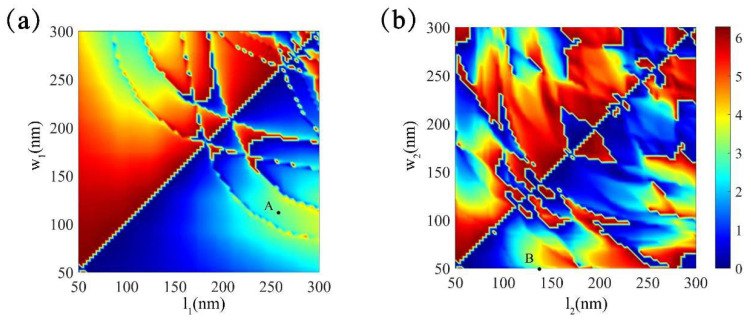
The phase shift difference (∆φ) of linearly polarized light along ordinary axes and extraordinary axes with different lengths of ordinary axes (l_1_, l_2_) and extraordinary axes (w_1_, w_2_) for (**a**) BTO nanofin and (**b**) Si nanofin.

The desired parameters of the BTO and Si nanofins were calculated by a three-dimensional FDTD method (Lumerical Inc., Vancouver, BC, Canada). The mesh grids were 10 nm × 10 nm × 10 nm. For the x- and y-axis, the periodic boundary condition was applied and the boundary condition for the z-axis was a perfectly matched layer (PML). The working wavelength was set to 600 nm to verify the proposed method. By changing the lengths of the ordinary axes (l_1_, l_2_) and the extraordinary axes (w_1_, w_2_) of the BTO nanofins (n_0_ = 2.4) and the Si nanofins with a period of 350 nm × 350 nm, the phase shift difference between the linearly polarized beam along the ordinary axes (x-polarized) and the extraordinary axes (y-polarized) can be seen in Figure 3. From Figure 3, points A and B with a phase shift difference π, which means the BTO nanofin (n_0_ = 2.4) and the Si nanofin can work as a half-wave plate, were the desired parameters. Therefore, parameters of the BTO nanofins and the Si nanofins were confirmed as l_1_ = 260 nm, w_1_ = 110 nm, H_1_ = 800 nm and l_2_ = 140 nm, w_2_ = 50 m, H_2_ = 600 μm, respectively. Then, the refractive index of BTO was changed to find a value that would meet the condition T_2_ = 0.

**Figure 4:**
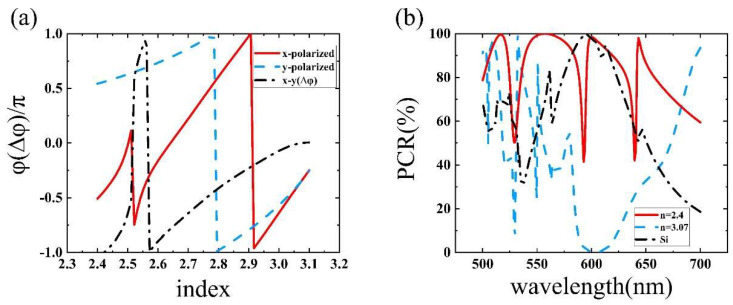
(**a**) Phase shift of the linearly polarized beam along ordinary axes (x-polarized) and extraordinary axes (y-polarized) and phase shift difference between them when the refractive index of the BTO nanofin changes from 2.4 to 3.1. (**b**) PCR of the BTO nanofin (*n* = 2.4 and *n* = 3.07) and the Si nanofin.

As shown in Figure 4a, for the BTO nanofins, the phase shift of the linearly polarized beam along the ordinary axes (x-polarized) and the extraordinary axes (y-polarized) and the phase shift difference between the output light of the two beams changed with the refractive index as depicted. The results showed that when the refractive index of the BTO layer was 2.4 (no applied voltage), the phase shift difference was π. During the period of increasing the refractive index to 3.07, the phase shift difference could cover all values from 0 − π, and become 0 when *n* = 3.07. In order to further show that the optimization results met the design requirements, the polarization conversion efficiency (PCR) of the BTO nanofins (*n* = 2.4 and 3.07) and the Si nanofins from 500 nm to 700 nm are depicted in Figure 4b. The results showed that the PCR of the BTO nanofins can reach 98.4% (*n* = 2.4) and 0.3% (*n* = 3.07) when the wavelength was 600 nm. Therefore, the resultant parameters can fit the desired functions well.

## 4. Results and Discussion

Based on all above analyses, a two-dimensional bifocal metalens was proposed. The phase profile to focus the incident beam had to meet the equation [34]:

(7) $$\varphi_{f}=\frac{2\pi }{\lambda }(f-\sqrt{x^{2}+y^{2}+f^{2}})$$

where λ is the working wavelength, f is the focal length and x and y are the discretized spatial coordinates. At last, in order to realize the proposed design, the rotation angle of the BTO nanofins and the Si nanofins should meet the equation as follows:

(8) $$\{\begin{array}{c} 2(\theta_{1}-\theta_{2})=\varphi_{f_{1}}\\ -2\theta_{2}=\varphi_{f_{2}} \end{array}$$

In the proposed bifocal metalens, the incident beam was right-handed circularly polarized. The wavelength was 600 nm. The radius of the metalens was set as 10 μm. To show the proposed design, we set the two focal lengths as f_1_ = 9 μm and f_2_ = 15 μm. The discussion of results is listed in Section 4.1 and Section 4.2

### 4.1. Switch of Single Focal Point

**Figure 5:**
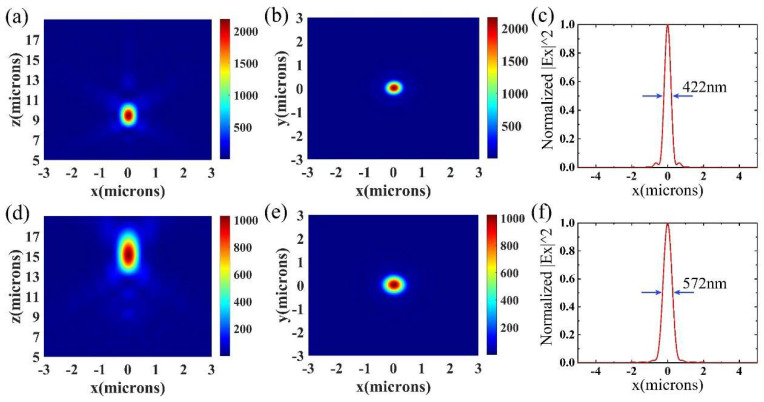
The simulated results for the refractive index of BTO was 2.4 and 3.07. (**a**,**d**) The intensity distribution of the first focal point (when *n* = 2.4) and the second focal point (when *n* = 3.07) on the x–z plane; (**b**,**e**) the intensity distribution of the first focal point and the second focal point on the x–y plane; (**c**,**f**) the full width at half-maxima of the first focal point (f_1_) and second focal point (f_2_) were 422 nm and 572 nm.

Firstly, in order to verify that the relative intensity of the two focal points can cover the extreme condition, we showed that the focal length of the metalens can switch between f_1_ and f_2_ in Figure 5. When there was no application of voltage, the refractive index of BTO was 2.4. The theoretical focal length was f_1_. After the voltage reached 60 V, the refractive index of BTO was 3.07. The theoretical focal length became f_2_. According to Figure 5a,d, the intensity profiles of the two conditions on the x–z plane were shown. The simulated focal lengths of BTO with *n* = 2.4 and *n* = 3.07 were 9.43 μm and 15.13 μm which were close to the design values. Figure 5b,e shows the intensity distribution of the first focal point and the second focal point on the x–y plane. Furthermore, in Figure 5c,f, to show the calculated spot size of the two focal points, we calculated the full width at half maximum (FWHM) to compare with the diffraction limited value according to the formula λ/2NA, where the equation of the numerical aperture NA was $NA=\sin\left[\tan^{-1}(D/2f)\right]$. When the diameter D of the metalens was 20 μm, the diffraction limited values of the two focal points were about 404 nm and 541 nm. The simulated FWHMs were 422 nm and 568 nm which corresponded to the theoretical diffraction limited values. For each focal point, on the focal plane, a circular area with a radius that is three times of its FWHM was picked. Then, the ratio of the optical power in this area to the power of the incident light was calculated to obtain the focusing efficiency [35]. The focusing efficiencies of the two focal points were about 46.60% and 40.15% when the refractive index was 2.4 and 3.07. The efficiency difference of the two foci may have mainly come from different numerical apertures (NAs).

### 4.2. Bifocal Metalens with Tunable Intensity Ratio

**Figure 6:**
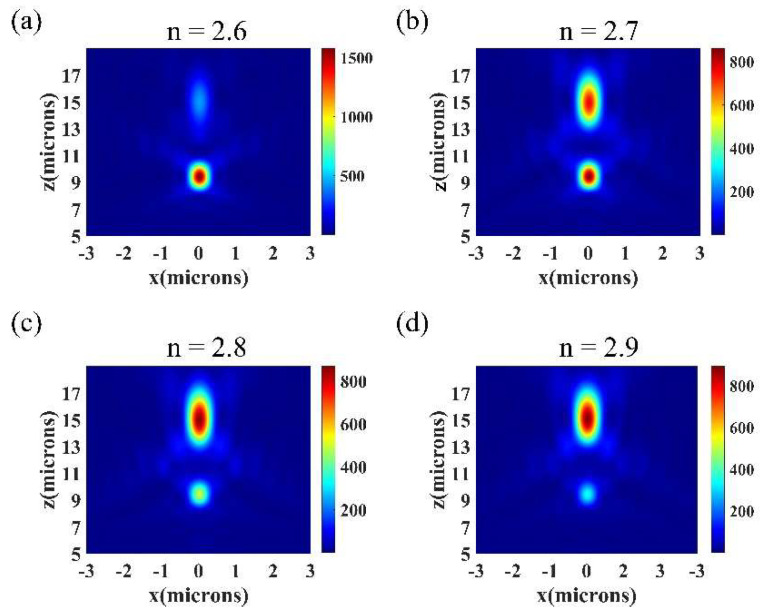
The intensity distribution of the bifocal metalens on the x–z plane when the refractive index of BTO was 2.6, 2.7, 2.8 and 2.9.

In order to demonstrate that the intensity ratio between the two focal points of the designed metalens can be modulated, the refractive index of BTO was set as 2.6, 2.7, 2.8 and 2.9 (with applied voltages of 18 V, 27 V, 36 V and 45 V). In Figure 6, the intensity distributions of the simulated results are shown. Combined with Figure 5a,d, the results showed that when the refractive index of BTO gradually increased, the intensity of the first focal point (with focal length f_1_ = 9 μm) would gradually decrease until the focal point disappeared. Additionally, the intensity of the second focal point (with focal length f_2_ = 15 μm) showed a trend of a gradual increase.

**Figure 7:**
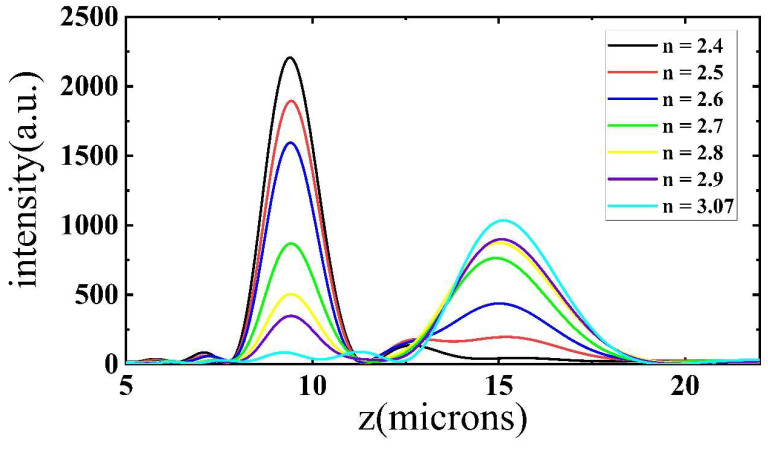
Simulated intensity results along z-axis when the refractive index of BTO changed.

Figure 7 shows the simulated results of the intensity along the z-axis (x = 0, y = 0). Additionally, Table 1 makes a summary of the focusing performance of the bifocal metalens with a different refractive index. The results show that the FWHM of each focal point was close to its theoretical diffraction limit. Additionally, the simulated focal lengths all met the designed values. Because for the one focal point case the position of the other focal point had no focal point, the FWHM and focal length were not calculated. Moreover, according to the intensity ratio, the ratio could reach 1/0.01 and 0.08/1 in the extreme condition with *n* = 2.4 and 3.07 (we chose the intensity at the theoretical focal point as the intensity of the disappearing focal point). The difference also came from the different NAs. The values were near the desirable values 1/0 and 0/1. When the refractive index increased from 2.4 to 3.07, the variation trend of the intensity ratio presented a process of gradual change, which means the design can alter the ratio flexibly.

## 5. Conclusions

In summary, we proposed a high-speed electrically modulated bifocal metalens with an adjustable intensity based on the EO material BTO in the visible region. The intensity ratio of the two focal points could be adjusted by applying different voltages of 0 to 60 V. When there was no application of voltage, the metalens could only focus on the first focal point (focal length is f_1_). As the applied voltage increased, the intensity of the first focal point gradually decreased. Meanwhile, the intensity of the second focal point (focal length is f_2_) increased with the voltage. Until the voltage reached 60 V, the design worked as a single-focusing metalens again with a focal length f_2_. Different from previous methods, the metalens realized through the intensity ratio between two different focal points can be adjusted arbitrarily with a constant optical source in the visible light band. In addition, when the voltage switched between 0 V and 60 V, the metalens could work as a varifocal metalens with two different focal lengths. The proposed electrically modulated metalens has great potential in the application of detectors, optical storage, laser printing and multi-functional devices.

## Figures and Tables

**Table 1:** The focusing performance of the bifocal metalens with different refractive index.

	*n* = 2.4	*n* = 2.5	*n* = 2.6	*n* = 2.7	*n* = 2.8	*n* = 2.9	*n* = 3.07
Simulated value (μm)	9.43	9.43	9.40	9.43	9.43	9.43	——
	——	15.20	15.02	14.93	15.00	15.09	15.13
FWHM (nm)	422	429	432	431	437	445	——
	——	563	564	568	563	567	568
Intensity ratio (f_1_: f_2_)	1/0.01	1/0.1	1/0.3	1/0.9	0.6/1	1/0.4	0.08/1

## Data Availability

No new data were created or analyzed in this study. Data sharing is not applicable to this article.
